# Incidence and risk factors for respiratory tract bacterial colonization and infection in lung transplant recipients

**DOI:** 10.1007/s10096-021-04153-1

**Published:** 2021-01-21

**Authors:** L. Paglicci, V. Borgo, N. Lanzarone, M. Fabbiani, C. Cassol, MG. Cusi, M. Valassina, S. Scolletta, E. Bargagli, L. Marchetti, P. Paladini, L. Luzzi, A. Fossi, D. Bennett, F. Montagnani

**Affiliations:** 1 Department of Medical Sciences, Infectious and Tropical Diseases Unit, Siena University Hospital, Siena, Italy; 2 Department of Medical and Surgical Sciences & Neurosciences, Respiratory Diseases and Lung Transplantation Unit, Siena University Hospital, Siena, Italy; 3 Department of Medical Biotechnologies, University of Siena, Siena, Italy; 4 Department of Innovation, Experimentation and Clinical Research, Microbiology and Virology Unit, Siena University Hospital, Siena, Italy; 5 Department of Emergency and Urgency, Medicine, Surgery and Neurosciences, Unit of Intensive Care Medicine, Siena University Hospital, Siena, Italy; 6 Cardio-Thoracic-Vascular Department, Anesthesia and Cardio-Thoracic-Vascular Intensive Care Unit, Siena University Hospital, Siena, Italy; 7 Cardio-Thoracic-Vascular Department, Thoracic Surgery Unit, Siena University Hospital, Siena, Italy

**Keywords:** Lung transplantation, Bacterial infections, Host bacteria interactions, Pulmonary fibrosis

## Abstract

To evaluate incidence of and risk factors for respiratory bacterial colonization and infections within 30 days from lung transplantation (LT). We retrospectively analyzed microbiological and clinical data from 94 patients transplanted for indications other than cystic fibrosis, focusing on the occurrence of bacterial respiratory colonization or infection during 1 month of follow-up after LT. Thirty-three percent of patients developed lower respiratory bacterial colonization. Bilateral LT and chronic heart diseases were independently associated to a higher risk of overall bacterial colonization. Peptic diseases conferred a higher risk of multi-drug resistant (MDR) colonization, while longer duration of aerosol prophylaxis was associated with a lower risk. Overall, 35% of lung recipients developed bacterial pneumonia. COPD (when compared to idiopathic pulmonary fibrosis, IPF) and higher BMI were associated to a lower risk of bacterial infection. A higher risk of MDR infection was observed in IPF and in patients with pre-transplant colonization and infections. The risk of post-LT respiratory infections could be stratified by considering several factors (indication for LT, type of LT, presence of certain comorbidities, and microbiologic assessment before LT). A wider use of early nebulized therapies could be useful to prevent MDR colonization, thus potentially lowering infectious risk.

**Supplementary Information:**

The online version contains supplementary material available at 10.1007/s10096-021-04153-1.

## Introduction

Lung transplant (LT) is considered a reasonable treatment option for selected patients with chronic respiratory end-stage diseases. The most frequent indications for adult LT are chronic obstructive pulmonary diseases (COPD) with or without α1-antitrypsin deficiency, interstitial lung diseases, cystic fibrosis, and idiopathic pulmonary arterial hypertension [1, 2]. Each one of these diseases has its own peculiar features in term of transplant list waiting, mortality, and post-transplant survival [3, 4].

Despite post-LT survival rate has increased over the last years, risk of early and late post-surgical complications remains high for transplanted patients [2, 5]. In particular, lung transplant recipients (LTRs) are at higher risk to develop infectious complications compared to other solid organ transplants; this can be due not only to immunosuppressive regimens and changes in anatomical structures after surgery, but also to constant exposure of airways to the outside environment [6, 7]. Infectious complications represent the most frequent cause of death after graft failure within the first 30 days from transplant, and the first cause within the first year [2].

Generally, the most common infections occur following a specific pattern based on the time from LT, as a consequence of the exposure to different risk factors over the time such as hospitalization, immunosuppression, reactivation of latent infection, and community-acquired pathogens exposure. During the early post-transplant period, infections can be related to post-surgical complications, donor or recipient’s pre-existing colonizations/infections, or nosocomial-acquired organisms [6–9]. Bacterial pneumonia is the most common infection in the first 30 days from LT, related to healthcare-associated or nosocomial bacteria. This risk decreases after 6 months from LT [7, 10] but the possibility to be colonized and infected by a multi-drug resistant (MDR) strain increases with the duration of pre-transplant hospitalizations and post-surgical intubation [1, 8].

Infections are also a major cause of late mortality, and a linkage (especially for bacterial aetiologies) with chronic rejection development is hypothesized, although their pathogenic role is not fully understood [1, 11].

Data on etiologic agents of pneumonia after lung transplant are limited for non-cystic fibrosis patients. Most of the bacteria isolated in post-transplant infections are Gram negative (e.g., *Pseudomonas aeruginosa*, *Klebsiella pneumoniae*) with a lower incidence of Gram positive bacteria, mainly represented by methicillin resistant *Staphylococcus aureus* (MRSA) [8, 12].

Prevalence and type of colonization, occurrence and type of infections and prevalence of MDR bacteria in the first month after transplantation can vary between patients in relation to the type of chronic illness causing end-stage organ disease, as well as to the clinical and microbiological history before and after transplantation [8, 13].

The aim of the study was to evaluate the incidence of and risk factors for respiratory bacterial colonization and infections within 30 days from LT in patients not affected by cystic fibrosis.

## Material and methods

### Patients

We retrospectively analyzed patients who underwent lung transplantation from January 1, 2001 to December 31, 2018 at the Lung Transplant Centre of the University Hospital of Siena, Tuscany, Central Italy. We included in the analysis only those subjects who had at least 18 years of age, had an available 1-month post-transplant follow-up, and complete clinical and microbiological data after transplantation. We excluded patients who underwent LT for cystic fibrosis since this disease has its own clinical features in terms of either recipient typology or pre-transplant colonization and infection etiologies, and therefore it is less comparable to the other lung transplant indications [14]. Patients were followed from the time of LT to a 1 month of follow-up and evaluated for the occurrence of bacterial respiratory colonization or infection, as below defined.

### Pre-transplant data

For each patient, demographic and clinical data were collected: gender, age, ethnicity, body mass index (BMI), indication for LT, type of LT (single/double), comorbidities (obesity, diabetes, arterial hypertension, dyslipidaemia, osteoporosis/osteopenia, peptic disease, cardiopathy, endocrinopathy, non-viral hepatopathy, psychiatric syndrome, nephropathy, rheumatic disease peripheral vasculopathy). We also evaluated previous hospitalizations occurring within 30 and 90 days before LT, administration of any antibiotic therapy in the pre-transplant period (≤ 7, 30, 90 days) and the use of respiratory devices (orotracheal intubation, tracheostomy) and/or extra-corporeal membrane oxygenation (ECMO) during the pre-transplant phase or during the surgery.

When retrospectively available, microbiological data from donors were also collected.

### Post-transplant data

The following post-transplant data were collected: presence of any devices (naso-gastric tube, NGT; central venous catheter, CVC; vesical catheter, VS; continuous veno-venous hemofiltration, CVVH; tracheostomy; orotracheal intubation; pleural drainage), length of hospital stay (LOS), occurrence of acute rejection, occurrence of primary graft dysfunction (PGD), and its grade (1-2-3) [15].

### Microbiological data

For each patient, we analyzed all respiratory samples [sputum, bronchoalveolar lavage (BAL) or bronchial aspirate (BAS)] that were collected for bacterial culture during the first month of follow-up post-LT. Bronchoscopy was regularly performed at day 1 after LT and every 24–48 h thereafter depending on clinical judgment, in case of symptoms or signs suggestive of lung infection and/or on the need to remove bronchial secretions. Bronchoalveolar lavage was always performed in association to transbronchial lung biopsy: all patients underwent surveillance biopsies at day 25–35 and thereafter on clinical demand. Airway samples, if collected during bronchoscopy, were routinely cultured.

Respiratory bacterial colonization was defined as isolation of bacteria from respiratory samples in the absence of significant laboratory and/or clinical alterations supporting diagnosis of infection. Respiratory bacterial infection was defined as isolation of bacteria from respiratory samples in the presence of significant laboratory and/or clinical alterations consistent with infection (e.g., fever, worsening cough and sputum production, dyspnoea, leucocytosis, increased C reactive protein, and/or procalcitonin).

For each bacterial isolate, we analyzed antimicrobial resistance profile, available from medical reports. The definition of multi-drug resistant (MDR), extensively drug resistant (XDR), pan-drug resistant (PDR) strain was based on the following criteria [16]: MDR was defined as acquired non-susceptibility to at least one agent in three or more antimicrobial categories, XDR was defined as non-susceptibility to at least one agent in all but two or fewer antimicrobial categories (i.e., bacterial isolates remain susceptible to only one or two categories) and PDR was defined as non-susceptibility to all agents in all antimicrobial categories.

### Prophylactic strategies

During the study period 2001 to 2018, our internal protocol (hereinafter, standard prophylaxis) consisted of universal prophylaxis with an extended spectrum cephalosporin, usually ceftazidime, plus a glycopeptide (vancomycin as first line therapy) started 1 h before the surgery and maintained for some days after surgery, depending on clinical judgment. Furthermore, prophylaxis with cotrimoxazole was usually started after 1 week to avoid the risk of *Pneumocystis jirovecii* pneumonia, in association with acyclovir universal prophylaxis. Finally, CMV prophylaxis was administered in case donor/recipient mismatch (D+/R-); in all other cases, a pre-emptive approach was adopted. Tailored approaches were used in case of donor or recipient’s previous bacterial isolates, based on the antimicrobial resistance profile. Use of voriconazole varied over the study years and on the basis of individual characteristics of patients, between universal prophylaxis and pre-emptive approach.

In case of previous isolates reporting susceptibility to polymixins (colistin), aerosol prophylaxis with this drug was prescribed in addition to systemic prophylaxis in selected patients. Furthermore, accordingly to our protocol, most patients received antimicrobial prophylaxis with nebulized taurolidine in the early post-transplant. This kind of strategy has been developed from the literature data available for patients transplanted for cystic fibrosis [17–20].

### Statistical analysis

Descriptive statistics [number, proportion, median, interquartile range (IQR)] were used to describe the baseline characteristics of patients. Categorical variables were compared by chi-square (*χ*^2^), Fisher exact test, and McNemar test when necessary. For the comparison of continuous variables, the Mann-Whitney *U* test was used. Factors associated to bacterial respiratory colonization and infection were analyzed by logistic regression. Only *p* values < 0.05 were considered to be statistically significant. All analyses were performed using the SPSS version 18.0 software package (SPSS Inc., Chicago, IL, USA).

## Results

### Population characteristics at baseline

Overall, 161 patients underwent LT during the study period. Thirty-one (19.5%) subjects were excluded since they were transplanted for cystic fibrosis, 36 (22.3%) were excluded for missing or incomplete clinical and/or microbiological data. Finally, a total of 94 patients fulfilled all the inclusion criteria and were included in the study. Table 1 summarizes their baseline characteristics. The studied population was mainly constituted by male (*n* = 59; 68.2%) patients with a median age of 56.5 years (IQR 49.8–61) and a median pre-transplant BMI of 24 kg/m^2^ (IQR 20–29). All the patients were Caucasian. Overall, 53.2% (*n* = 50) of recipients had an optimal BMI (18.5–24.99 kg/m^2^) in pre-transplant period, 41.5% were overweighed (BMI 25–29.99 kg/m^2^), or mildly obese (BMI 30–34.99 kg/m^2^) (respectively, *n* = 19 and *n* = 20). A percentage of 5.3 (*n* = 5) of patients were classified as underweighted during the pre-transplant evaluation and no patient had a baseline BMI greater than 33 kg/m^2^.

**Table 1:** Baseline characteristics of the study population (*n* = 94)

Characteristics	*N* (%) or median (IQR)
Age, years	56.5 (49.8–61)
Male:	59 (62.8)
Pre-transplant BMI, kg/m^2^	24 (20–29)
Indication for transplant:	
- IPF	41 (43.6)
- COPD	24 (25.5)
- Other	29 (30.9)
Comorbidities:	78 (83)
- Osteoporosis/osteopenia	48 (51.1)
- Diabetes	23 (24.5)
- Mild obesity (BMI ≥ 30)	20 (21.3)
- Arterial hypertension	20 (21.3)
- Peptic disease	11 (11.7)
- Chronic coronary heart disease	9 (9.6)
- Dyslipidaemia	8 (8.5)
- Psychiatric disorder	8 (8.5)
- Endocrinopathy	7 (7.4)
- Non-viral hepatopathy	7 (7.4)
- Peripheral vasculopathy	7 (7.4)
- Rheumatic autoimmune disease	4 (4.3)
- Nephropathy	3 (3.2)
- Other	32 (34)
Hospitalization 90 days before LT	20/90 (22.2)
Antibiotic treatment 90 days before LT	17/86 (19.8)
ICU admission 90 days before LT	5/86 (4.7)
Type of planned LT	
- Single	52 (53.4)
- Double	42 (44.6)
Baseline respiratory bacterial colonization (recipient)	
- Overall	13 (13.8)
- MDR bacteria	2/90 (2.2)
Baseline respiratory bacterial infection (recipient)	
- Overall	6/90 (6.6)
- MDR bacteria	1/90 (1.1)
Baseline respiratory bacterial colonization (donor)	
- Overall	23/84 (27.4)
- MDR bacteria	2/80 (2.5)
Baseline respiratory bacterial infection (donor)	
- Overall	6/81(7.4)
- MDR bacteria	0/77(0)

*BMI*, body mass index; *IPF*, idiopathic pulmonary fibrosis; *COPD*, chronic obstructive pulmonary disease; *LT*, lung transplant; *ICU*, intensive care unit; *MDR*, multi-drug resistant

The most common indication for LT was idiopathic pulmonary fibrosis (IPF) (*n* = 41; 43.6%), followed by COPD (*n* = 24; 25.5%). Twenty-nine patients (30.8%) underwent LT for other indications, notably interstitial lung diseases different from IPF in 55% of the remaining cases (*n* = 16/29) (e.g., non-specific interstitial pneumonia, NSIP; allergic extrinsic alveolitis; interstitial diseases in patients with systemic sclerosis and autoimmune arthritis; graft versus host disease), sarcoidosis (*n* = 3/29, 10.3%), pulmonary microlitiasis (*n* = 3/29, 10.3%), pulmonary Langerhans cell histiocytosis (*n* = 2/29, 6.9%), non-CF bronchiectasis (*n* = 2/29, 6.9%), lymphangioleiomyomatosis (*n* = 1/29, 3.4%), primary ciliary dyskinesia (*n* = 1/29, 3.4%), obliterans bronchiolitis (*n* = 1/29, 3.4%).

On the total, 78 patients (83%) had at least one comorbidity. The most common was osteoporosis/osteopenia (*n* = 48; 51.1%), followed by diabetes (*n* = 23, 24.5%), obesity (*n* = 20, 21.3%), arterial hypertension (*n* = 20, 21.3%); 11.7% of patients had peptic diseases (gastroesophageal reflux, gastritis or peptic ulcer). Most of patients underwent single lung transplantation (55.3%, *n* = 52/94).

During the pre-transplant screening evaluation, 13.8% of recipients (*n* = 13) had a bacterial respiratory colonization, with a small prevalence of MDR strains (*n* = 2/90 available reports, 2.2%). Respiratory infections were found in 6.6% of cases (*n* = 6), with one case of infection by MDR bacteria. Available retrospective microbiological data from donors showed 27.4% (*n* = 23/84) cases of colonization and 7.4% (*n* = 6/81) cases of infection. On the basis of accessible microbiological results, only 2.5% (*n* = 2/80) of isolates from colonized donors were MDR bacteria; no MDR bacteria were revealed from infected donors.

On the basis of available data, 22.2% (*n* = 20/90) of the recipients had been hospitalized within 90 days before surgery, of whom 17.1% (*n* = 15/88) in the previous 30 days. A limited percentage of the population (*n* = 5/86, 5.8%) had been admitted to intensive care units (ICU) during pre-LT hospitalization. Antibiotic therapy had been prescribed in a limited number of patients within 90 days preceding the transplant (*n* = 17/86, 19.8%), mostly in the week before the transplant (*n* = 12/79, 15.2%).

#### Post-transplant follow-up

After surgery, ventilation was administered initially by endotracheal tube in all cases. The mean time of intubation was 96 h (IQR 48–168 h). In 23.3% (*n* = 20/86) of patients, a tracheostomy was placed during the hospitalization. Twenty-four of 85 patients (38.2%) needed surgical revision, after a median time of 11.5 days (IQR 6.5–26.3) from transplant.

Acute rejection was observed in 26 cases (27.7%), after a median time of 23.5 days from the surgery (IQR 17–36.3 days). On the total of available histologic referrals, 34.6% of patients (*n* = 9) were classified as grade 1, 38.4% (*n* = 10) as grade 2, 3.8% as grade 3 (*n* = 1), 3.8% as grade 4 (*n* = 1). A percentage of 7.6 (*n* = 2/26) had a humoral rejection.

PGD occurred in 69% of cases (*n* = 64), mostly grade 2 (32.3%). During the study period 83% (*n* = 78) underwent a standard systemic antibiotic prophylaxis regimen, while the remaining 17% (*n* = 16) received a different scheme of prophylaxis based on previous microbiological isolates and/or allergies. Mean duration over the study period was 15 days (IQR 11–18) for anti-Gram negative agents and 12 days (IQR 8–18) for anti-Gram positive drugs. Overall, 87.2% (*n* = 82) received aerosol prophylaxis after the transplant, for a mean duration of 29 days (IQR 20–43 days). Of this group, 89% (*n* = 73/82) received taurolidine as aerosol prophylaxis, for a mean time of 30 days (IQR 21.5–45 days).

#### Bacterial respiratory colonization after transplantation

Thirty-three percent of the population (*n* = 31) developed bacterial colonization of the lower respiratory airways within the first month from LT. On the total of patients with available antimicrobial susceptibility test of post-LT isolates, 11.4% (*n* = 10/88) were colonized by MDR bacteria and one by an XDR bacterium. No PDR isolates were found. The most frequent MDR isolates after the transplant were *Stenotrophomonas maltophilia* (*n* = 4), MRSA (*n* = 3), *Enterococcus fecalis* (*n* = 2). One XDR *Klebsiella pneumoniae* was isolated, susceptible only to gentamicin and colistin.

A significant increase in overall and MDR/XDR bacterial colonization was revealed comparing pre- and post-LT period: respectively, 13.8% (*n* = 13/94) versus 33% (*n* = 31/94), *p* < 0.0001 and 2.2% (*n* = 2/88) versus 12.5% (*n* = 11/88), *p* < 0.0001.

Differences between patients showing bacterial respiratory colonization (all bacteria and MDR bacteria) during the first month after transplantation are showed in Table 2. Patients with overall bacterial respiratory colonization had most probably received bilateral LT (61.3% vs. 36.5% of those not colonized, *p* = 0.02). On the other side, subjects colonized by MDR had more frequently peptic diseases (40% vs. 7.7% in those not colonized by MDR, *p* = 0.01) and a shorter length of aerosol prophylaxis (29 days vs. 17 days, *p* = 0.01).

**Table 2:** Risk factors for bacterial respiratory colonization 30 days after LT

Risk factors	30-day colonization *N* = 31 (%)	No 30-day colonization *N* = 63 (%)	*p* value	30-day MDR colonization *N* = 10 (%)	No 30-day MDR colonization *N* = 78 (%)	*p* value
Males	21 (67.7)	38 (60.3)	0.48	7 (70)	29 (37.2)	0.74
Median age (IQR)	55 (49.61)	58 (52–62)	0.16	59 (54.8–64.3)	48.5 (47.8–52)	0.29
Pre-LT BMI	24 (20–30)	24 (20–28)	0.65	25.4 (20–30.5)	24 (20–27.3)	0.30
Indication for transplant:			0.72			0.07
- IPF - COPD - Other	15 (48.4) 8 (25.8) 8 (25.8)	26 (41.3) 16 (25.4) 21 (33.3)		8 (80) 1 (10) 1 (10)	32 (41) 20 (25.6) 26 (33.3)	
Type of transplant:			**0.02**			0.51
- Single - Double	12 (38.7) 19 (61.3)	40 (63.5) 23 (36.5)		7 (70) 3 (30)	43 (55.1) 35 (44.9)	
Comorbidities: - Osteoporosis/osteopenia	26 (83.9) 18 (58.1)	52 (82.5) 30 (47.6)	0.87 0.34	8 (80) 5 (50)	65 (83.3) 38 (48.7)	0.68 0.94
- Peptic disease	6 (19.4)	5 (7.9)	0.11	4 (40)	6 (7.7)	**0.01**
- Chronic coronary heart disease	6 (19.4)	3 (4.8)	0.06	2 (20)	7 (9)	0.27
- Diabetes	7 (22.6)	16 (25.4)	0.77	3 (30)	19 (24.4)	0.71
- Obesity	8 (25.8)	12 (19)	0.45	4 (40)	14 (17.9)	0.20
- Nephropathy	1 (3.2)	2 (3.2)	0.42	0 (0)	2 (2.6)	1
Hospitalization 90 days before LT	6 (19.4)	14/59 (23.7)	0.32	2/10 (20)	17/74 (23)	0.68
ICU admission 90 days before LT	6 (19.4)	3/57 (5.3)	0.82	0/9 (0)	4/72 (5.5)	0.75
Antibiotic treatment 90 days before LT	3/30 (10)	14/56 (25)	0.11	1 (10)	16/71 (22.5)	0.39
Baseline respiratory bacterial colonization (recipient):						
- Overall	4 (12.9)	9 (14.3)	0.81	2 (20)	10 (12.8)	0.62
- MDR bacteria	0/29 (0)	2/61 (3.3)	0.47	0/9 (0)	2/76 (2.6)	0.14
Baseline respiratory bacterial infection (recipient):						
- Overall	2 (6.5)	4/59 (6.7)	0.34	1 (10)	5/74 (6.8)	0.54
- MDR bacteria	0 (0)	1/59 (1.7)	0.63	0/9 (0)	1/74 (1.4)	0.77
Baseline respiratory bacterial colonization (donor):						
- Overall	10/28 (35.7)	13/56 (23.3)	0.24	2/8 (25)	18/70 (25.7)	1
- MDR bacteria	1/26 (3.8)	1/50 (1.9)	0.84	0/8 (0)	2/67 (3)	1
Baseline respiratory bacterial infection (donor):						
- Overall	0/27 (0)	6/54 (11.1)	0.19	0/8 (0)	6/67 (9)	1
- MDR bacteria	0/27 (0)	0/50 (0)	0.36	0/8 (0)	0/63 (0)	1
Post-LT systemic antibiotic prophylaxis						
-Standard^a^						
-Atypical	23 (74.2) 8 (25.8)	55 (87.3) 8 (12.7)	0.11	7 (70) 3 (30)	67 (85.9) 11 (14.1)	0.194
Aerosol prophylaxis length of days median (IQR)	27 (87.1) 25 (17–32)	55 (87.3) 29 (21–44)	0.98 0.14	10 (100) 17 (6.5–31.5)	66 (84.6) 29 (22–44.5)	0.35 **0.01**
Basiliximab induction	14 (45.2)	20 (31.7)	0.20	3 (30)	7 (34.6)	1
Immunosuppressive regimen:						
- Tacrolimus - Cyclosporine - Other	11/28 (39.3) 17/28 (60.7) 0/28 (0)	11/55 (39.3) 17/55 (60.7) 2/55 (3.6)	0.53	3/9 (33.3) 6/9 (66.7) 0/9 (0)	41/68 (60.3) 25/68 (36.8) 2/68 (2.9)	0.84
Rejection Days from LT, median (IQR) PGD	9 (29) 24 (18.5–38.5) 21 (67.7)	17 (27) 22 (17–37) 43 (69.4)	0.84 0.69 0.87	3 (30) 43 (20–50) 7 (70)	20 (25.6) 22.5 (16.5–34.8) 51/77 (66.2)	0.72 0.30 1
Lenght of stay, days median (IQR)	36 (28–60)	37 (29–66)	0.76	36.5 (28–61.3)	55.5 (27.6–75.5)	0.40

*BMI*, body mass index; *IPF*, idiopathic pulmonary fibrosis; *COPD*, chronic obstructive pulmonary disease; *LT*, lung transplant; *MDR*, multi-drug resistant; *PGD*, primary graft dysfunction; ^a^comprehending one of each of the following classes: anti-pseudomonal cephalosporin (namely ceftazidime), anti-Gram positive drug (namely vancomycin), antifungal prohylaxis (voriconazole), and trimethoprim-sulfamethoxazole prophylaxis

Factors associated to bacterial colonization (both overall and by MDR strains) during the first month post-LT were investigated by logistic regression; results are summarized in Table 3. Bilateral LT [adjusted odd ratio (AOR) 3.61, 95% confidence intervals (CI) 1.35–9.60, *p* = 0.01] and chronic coronary heart disease (AOR 6.67, 95% CI 1.36–32.82 *p* = 0.02) were independently associated to a higher risk of overall bacterial colonization. On the other side, peptic diseases (AOR 7.66, 95% CI 1.48–39.77, *p* = 0.01) was associated with a higher risk of colonization by MDR strains, while those with longer duration of aerosol prophylaxis showed a lower risk of MDR colonization (AOR 0.95 per 1 day increase, 95% CI 0.90–1.00, *p* = 0.04).

**Table 3:** Risk factors for respiratory bacterial colonization at 30-day post-LT (logistic regression)

Univariate			Multivariate			
	OR	CI 95%	*p* value	aOR	CI 95%	*p* value
Overall 30-day colonization						
Double LT	2.75	1.11–6.68	**0.02**	3.61	1.35–9.60	**0.01**
Chronic coronary heart disease	4.80	1.11–20.72	**0.04**	6.67	1.36–32.82	**0.02**
MDR 30 days colonization						
Peptic disease	8	1.76–36.38	**0.007**	7.66	1.48–39.77	**0.01**
Aerosol prophylaxis, mean time, per 1-day increase	0.95	0.91–1.00	**0.046**	0.95	0.90–1.00	**0.04**

*LT*, lung transplant; *MDR*, multi-drug resistant bacteria

A further analysis comparing patients not colonized versus those colonized by susceptible strains versus those colonized by MDR/XDR bacteria is provided as supplementary material, confirming peptic diseases as a risk factor for 30 days MDR colonization (Supplementary Table 1).

#### Bacterial respiratory infections after transplantation

Overall, 35.5% of lung recipients (*n* = 33) developed bacterial pneumonia, and in more than half of the cases (*n* = 19/33; 57.6%) MDR bacteria were the responsible agents. The main MDR strains isolated in case of pneumonia were MRSA (*n* = 6), *Pseudomonas aeruginosa* (*n* = 5), *Enterococcus faecium* (*n* = 2), *E. coli* (*n* = 2), *Acinetobacter baumannii* (*n* = 1). No XDR pathogen was isolated during infections in the post-LT observed period.

Differences between patients showing bacterial respiratory infection (all bacteria and MDR bacteria) during the first month after transplantation are showed in Table 4. Patients developing pneumonia in the early post-transplant period had more frequently a history of previous infection in the pre-transplant period (21.2% vs. 0%, *p* = 0.01) and a longer length of hospital stay (median 51 vs. 34 days, *p* = 0.03). Pre-LT diagnosis of IPF was a significant risk factor for developing both MDR (*p* < 0.01) and non-MDR infections (*p* = 0.03). In MDR infected population a higher prevalence of PGD (all grades) was reported: 94.7% of recipients (*n* = 18/19) vs. 63.4% (*n* = 45/72) of those not MDR infected, *p* = 0.01.

**Table 4:** Risk factors for bacterial respiratory infection 30-days after LT

Risk factors	30-day infection *N* = 33 (%)	Not infected *N* = 60 (%)	*p* value	30-day MDR infection *N* = 19 (%)	Not MDR infected *N* = 72 (%)	*p* value
Males	17 (51.5)	41 (68.3)	0.11	11(57.9)	46 (63.9)	0.63
Median age (IQR)	57 (48.5–61.5)	56 (51–61)	0.70	60 (55–63)	56 (49–60.8)	0.10
Pre-transplant BMI	23 (20–25.7)	24.4 (20.1–30)	0.53	24 (20–27)	24 (20–29)	0.98
Indication for transplant:			**0.03**			**< 0.01**
- IPF - COPD - Other	20 (60.6) 4 (12.2) 9 (27.3)	21 (35) 19 (31.7) 20 (33.3)		14 (73.7) 1 (5.3) 4 (21.1)	26 (36.1) 22 (30.6) 24 (33.3)	
Type of transplant:			0.81			0.26
- Single - Double	19 (57.6) 14 (42.4)	33 (55) 27 (45)		13 (68.4) 6 (31.6)	39 (54.2) 33 (45.8)	
Comorbidity: - Osteoporosis/osteopenia	25 (75.8) 14 (42.4)	52 (86.7) 33 (55)	0.18 0.25	15 (78.9) 8 (42.1)	61 (84.7) 38 (52.8)	0.51 0.41
- Peptic disease	5 (15.2)	6 (10)	0.46	4 (21.1)	7 (9.7)	0.23
- Chronic coronary heart disease	3 (9.1)	6 (10)	1	2 (10.5)	7 (9.7)	1
- Diabetes	6 (18.2)	17 (28.3)	0.28	5 (26.3)	18 (25)	0.91
- Obesity	4 (12.1)	16 (26.7)	0.12	4 (21.1)	16 (22.2)	1
- Nephropathy	2 (6.1)	1 (1.7)	0.25	1 (5.3)	2 (2.8)	0.51
Hospitalization 90 days before LT	9 (27.3)	11 (19.6)	0.41	5 (26.3)	14 (20.6)	0.59
ICU admission days before LT	1 (3)	3 /52 (5.8)	1	0 (0)	4/64 (6.3)	0.16
Antibiotic treatment 90 days before LT	5/31 (16.1)	12/54 (22.2)	0.64	3/17 (17.6)	13/66 (19.7)	0.94
Baseline respiratory bacterial colonization (recipient):						
- Overall	7 (21.2)	7 (11.7)	0.22	6 (31.6)	8 (11.1)	**0.03**
- MDR bacteria	2/31 (6.5)	0/58 (0)	0.12	2/18 (11.1)	0/69 (0)	**0.04**
Baseline respiratory bacterial infection (recipient):						
- Overall	7 (21.2)	0/56 (0)	**< 0.01**	4/19 (21.1)	2/68 (2.9)	**0.02**
- MDR	1/32 (3.1)	0/56 (0)	0.31	1/18 (5.6)	0/68 (0)	0.15
Baseline respiratory bacterial colonization (donor):						
- Overall	8/32 (25)	15/51 (29.4)	0.66	6 (31.6)	17/62 (27.4)	0.73
- MDR bacteria	1/32 (3.1)	1/47 (2.1)	1	1 (5.3)	1/58 (1.7)	0.44
Baseline respiratory bacterial infection (donor):						
- Overall	3/31 (9.7)	3/49 (6.1)	0.67	2/18 (11.1)	4/60 (6.7)	0.62
- MDR bacteria	0/29 (0)	0/47 (0)	0.40	3 (15.8)	14 (23.3)	1
Systemic antibiotic prophylaxis:						
- Standard^a^ - Atypical	24 (72.7) 9 (27.3)	53 (88.3) 7 (11.7)	**0.056**	13 (68.4) 6 (31.6)	62 (86.1) 10 (13.9)	**0.072**
Aerosol prophylaxis length of days median (IQR)	32 (97) 32(21.3–46.8)	49 (81.7) 25(18–40.5)	0.05 0.15	19 (100) 33 (22–51)	60 (83.8) 28.5(18.5–41.8)	0.07 0.19
Basiliximab induction	13 (39.4)	20 (33.3)	0.56	6 (31.6)	26/72 (36.1)	0.71
Immunosuppressive regimen:						
**- **Tacrolimus - Cyclosporine - Other	10/29 (34.5) 19/29 (65.5) 0/29 (0)	18/53 (34) 33/53 (62.3) 2/53 (3.8)	0.57	6/16 (37.5) 10/16 (62.5) 0/16 (0)	21/64 (32.8) 41/64 (64.1) 2/64 (3.1)	0.74
Rejection	10 (30.3)	16 (26.7)	0.71	7 (36.8)	19 (26.4)	0.37
Days from LT, median (IQR)	22 (12.8–38.5)	24 (19.3–34.8)	0.55	23 (17–43)	24 (16–35.5)	0.80
PDG	27 (81.8)	37 (62.7)	0.06	18 (94.7)	45 (63.4)	**0.01**
Length of stay, days median (IQR)	51(32–74)	34.5 (27–54.8)	**0.03**	51 (33–70)	35.5 (28–56.5)	0.15

*BMI*, body mass index; *IPF*, idiopathic pulmonary fibrosis; *COPD*, chronic obstructive pulmonary disease; *LT*, lung transplant; *MDR*, multi-drug resistant; *PGD*, primary graft dysfunction; ^a^comprehending one of each of the following classes: anti-pseudomonal cephalosporin (namely ceftazidime), anti-Gram positive drug (namely vancomycin), antifungal prophylaxis (voriconazole), and trimethoprim-sulfamethoxazole prophylaxis

Factors associated to bacterial infection (both overall and by MDR strains) during the first month post-LT were investigated by logistic regression; results are summarized in Table 5. COPD (AOR 0.17 versus IPF, 95% CI 0.05–0.66, *p* = 0.01) and higher BMI (AOR 0.87 per 1 kg/m^2^ increase, 95% CI 0.78–0.98, *p* = 0.03) were associated to a lower risk of bacterial infection, while aerosol prophylaxis with taurolidine was associated to a higher risk (AOR 5.86 versus no aerosol prophylaxis, 95% CI 1.05–32.69, *p* = 0.04). Narrowing the analysis to MDR infected patients, patients with IPF confirmed a higher risk of infection (*p* < 0.05, see Table 5); a higher risk of MDR infection was also observed in those with pre-transplant colonization (AOR 5.37, 95% CI 1.02–28.28, *p* = 0.04) and pre-transplant infections (AOR 16.84, 95% CI 1.18–239.39, *p* = 0.01).

**Table 5:** Risk factors for respiratory bacterial infection at 30-day post-LT (logistic regression)

	Univariate				Multivariate	
	OR	95% CI	*p* value	aOR	95% CI	*p* value
30-day overall infection						
Transplant indication						
- COPD vs. IPF - Others vs. IPF	0.22 0.47	0.06–0.76 0.17–1.28	**0.02** 0.14	0.17 0.52	0.05–0.66 0.17–1.59	**0.01** 0.25
BMI (per 1 kg/m^2^ increase)	0.90	0.81–1.00	**0.049**	0.87	0.78–0.98	**0.03**
Aerosol						
- No aerosol prophylaxis	REF	REF	REF	REF	REF	REF
- Taurolidine aerosol	4.5	0.95–21.38	**0.06**	5.86	1.05–32.69	**0.04**
- Taurolidine + other antibiotic	3.5	0.21–51.77	0.38	5.72	0.29–110.7	0.24
Antibiotic aerosol in addition to systemic therapy	10.5	1.03–107.17	**0.047**	10.32	0.81–130.33	0.07
Atypical^a^ prophylaxis	2.83	0.94–8.52	0.06	2.27	0.64–8.07	0.20
30-days MDR infection						
Transplant indication:						
- COPD vs. IPF	0.08	0.01–0.84	**0.02**	0.07	0.01–0.77	**0.03**
- Other vs. IPF	0.31	0.08–1.10	**0.06**	0.17	0.03–0.86	**0.03**
Pre-LT respiratory colonization	3.69	0.78–16.88	**0.03**	5.37	1.02–28.28	**0.04**
Pre-LT respiratory infection	8.8	0.59–1.40	**0.02**	16.84	1.18–239.39	**0.04**
PGD	10.4	1.13–82.48	**0.03**	6.45	0.75–56.37	0.09
Atypical^a^ prophylaxis	2.86	0.88–9.27	0.08	1.83	0.34–9.92	0.48

*CODP*, chronic obstructive pulmonary disease; *IPF*, interstitial pulmonary fibrosis; *BMI*, body mass index; *PGD*, primary graft dysfunction; *REF*, reference for risk factors analysis; *MDR*, multi-drug resistant; ^a^not comprehending at least one of the following: anti-pseudomonal cephalosporins (mainly ceftazidime), anti-Gram positive drug (mainly vancomycin), antifungal (mainly voriconazole), or trimethoprim-sulfamethxazole prohylaxis

Both baseline bacterial colonization and baseline respiratory infections were statistically related to MDR respiratory infections within 30 days after LT (see Table 5). Specifically, in 1/2 (50%) pre-LT MDR colonized patients and in 1/11 (9.1%) pre-LT non-MDR colonized patients, the same bacteria were responsible of post-LT infections, respectively MDR *S. aureus* and non-MDR *S. marcescens*. Moreover, 2/5 (40%) patients with pre-LT non-MDR infection developed post-LT infection by the same bacteria (1 *E. coli* and 1 *P. aeruginosa*). The only one patient with pre-LT pneumonia sustained by MDR *P. aeruginosa* was colonized by the same bacterium in the post-LT BAL specimens but did not develop an infection.

On the total of 31 post-LT colonized patients, 5/10 (50%) MDR colonized and 6/21 (28.6%) non-MDR colonized patients developed infections: in 2 cases, infection was caused by the same colonizing bacteria (respectively 1 MDR *S. aureus* and 1 non-MDR *E. faecalis*).

#### Immunosuppressive regimens and microbiological outcomes

All patients received corticosteroid therapy consisting of intravenous methylprednisolone 125 mg before graft reperfusion followed by 375 mg on day 0 and tapering from 1 mg/kg on day 1. Induction therapy was introduced in our protocol in 2009; however, since then not all patients received it, depending on the decision of the surgeon.

One-third of patients (36.2% *n* = 34/94) were treated with basiliximab (20 mg on day 0 and day 4); only a few (2.6%, *n* = 2/94) received rabbit antithymocyte globulin (1.5 mg/kg/day for 3–5 days).

Calcineurin inhibitors were introduced on days 3–5 using either tacrolimus (trough level 10–15 ng/ml before introduction of basiliximab, 8–10 ng/ml thereafter) or cyclosporine (trough level 250–300 ng/ml before introduction of basiliximab, 200–250 ng/ml thereafter). Cyclosporine was used predominantly until 2007; tacrolimus was used thereafter. Azathioprine 100 mg/day or mycophenolate mofetil 1 g/day was introduced in most patients from day 7 to day 10. In 2007, mycophenolate mofetil replaced azathioprine in the baseline regimen for all patients.

Rates of colonization or infection (both globally and by MDR) at 30 days post-LT were not significantly different when comparing subgroups treated with different immunosuppressive regimens (cyclosporine versus tacrolimus versus all other regimen) or subgroups with or without basiliximab induction (see Tables 2 and 4).

#### Antibiotic prophylaxis schemes and microbiological outcomes

Patients treated with standard systemic antibiotic prophylaxis did not show significantly different 30 days post-LT bacterial colonization rates when compared to those receiving alternative regimens [overall colonization: 29.5% (23/78) versus 50% (8/16), *p* = 0.112; MDR colonization: 9.5% (7/74) versus 21.4% (3/14), *p* = 0.194].

Higher rates of 30-days post-LT bacterial infection were reported in the subgroup of patients receiving an alternative prophylaxis scheme when compared to those treated with standard prophylaxis [overall infections: 56.3% (9/16) versus 31.2% (24/77), *p* = 0.056]. Infections by MDR bacteria were diagnosed in 17.3% (13/75) of patients receiving standard prophylaxis regimen versus 37.5% (6/16) of patients receiving an alternative scheme (*p* = 0.072). However, these trends toward an association were not confirmed in multivariate analyses (see Table 5).

No significant differences regarding colonization and infection rates (overall and by MDR) were observed between patients receiving aerosolized antibiotics versus those not receiving them (data not shown).

## Discussion

Graft colonization and infection in lung transplant recipients represent a common finding [13], with a higher risk observed especially in the first months after surgery [8]. In early post-surgery, the lung allograft is particularly susceptible to infections: the epithelium dysfunction related to the disruption of bronchial circulation and lymphatic drainage, the denervation of the allograft causing a loss of cough reflex and bronchial hyperresponsivity, and the possible stenosis or necrosis of the bronchial anastomosis may facilitate colonization or infection of airways mucosae and/or lung tissue [7]. Infections are the most common cause of death in lung recipients [1]. Most of the related literature on post-transplant infection management is based on experts’ opinion and single center retrospective studies, especially in Europe, but a comprehensive global analysis is difficult to achieve. Every day clinical management in different lung transplant centers may differs due to epidemiological peculiarity [21] and local evidence based medicine. Results from each single center could enrich data analysis and offer confrontation for clinical management.

With the limitations of a retrospective specific cohort analysis, our study revealed a significant increase of respiratory bacterial colonization by both overall bacteria (*p* < 0.0001) and by MDR/XDR bacteria (*p* < 0.0001) in the 30 days post-LT. A significantly greater percentage of 30-days respiratory infections occurred in patients with a baseline bacterial colonization (*p* = 0.03) or infection (*p* = 0.02).

Donor-derived infections are also of major concern [9]; however, a low risk of donor-recipient MDR transmission has been previously hypothesized [22]. In our cohort, at least 31% of donors had a respiratory bacterial colonization, with a low percentage of MDR bacteria (2.5%), but these observations appeared not to be related to colonization and infection in recipients.

In our cohort, the transplant type seemed to play a role in the prediction of post-transplant infectious risk within the first month after LT. In fact, patients with 30 days pulmonary colonization most frequently received baseline double lung transplant (61.3% vs. 36.5%, *p* = 0.02). Discordant data are reported in literature regarding correlation between type of LT, infectious complications, and outcome. Meyer et al. observed that single lung transplant in patients with IPF had a better 30-day and 1-year outcome, although the reasons were not clarified, and a role of early events (e.g., infections and colonizations together with surgical technical procedure) was hypothesized [23]. Other studies suggested that bilateral LT did not differ from single LT in terms of overall mortality, even if single LT could have better rates of long-term survival after transplant in > 60-year-old recipients [24, 25] and, on the other hand, that bilateral LT can offer a survival advantage in younger recipients with end-stage emphysema [24, 26]. Our findings can suffer from biases of a retrospective study; however, reported discordant data underline the need of further analysis regarding correlation of type of LT and infectious risk.

We also found that patients with chronic coronary heart disease were more subject to develop lower respiratory tract bacterial colonization in the first month after surgery. Twenty percent of lung recipients had ischemic heart disease as a baseline comorbidity, and these patients had a sixfold increment of bacterial colonization.

Our results showed also that peptic disease (non-ulcerative gastritis; esophagitis; gastric ulcer; and gastroesophageal reflux disease, GERD) was associated to a higher risk of MDR bacteria colonization in the first month after LT. This finding is in line with previous reports [27, 28], which also highlights a high incidence of peptic disease in lung recipients and a more rapid progression to acute and chronic graft rejection after transplant in patients affected by this condition. This can be theoretically explained by various factors, such as continuous asymptomatic aspiration of peptic material which can lead to direct injuries and trigger inflammatory response within the graft, thus facilitating bacterial colonization.

Interestingly, we also found that duration of nebulized therapy was linked to the risk of MDR colonization. In particular, patients who underwent longer periods of aerosolized antibiotic drugs administration were found to be less prone to develop lung MDR colonization within the first 30 days. No benefits were found about the use of aerosolized taurolidine (a topic decontaminant used in many lung transplant centers) as a prophylactic strategy; indeed, aerosolized taurolidine was associated to a higher risk of bacterial infections. This finding should be interpreted with caution due to the small study population and retrospective design of the study, since taurolidine could have been prescribed to patients at higher risk to develop infections: in our center, taurolidine aerosol is indeed commonly used, in combination with systemic antibiotics, as salvage off label therapy in severe respiratory infections after LT. As a consequence, prospective randomized studies are needed to clarify the role of taurolidine as an effective agent used to prevent bacterial respiratory infections. Nebulized administration of antibiotic can be a helpful additional strategy to decontaminate airways after transplant, especially from Gram negative isolates [29], although data from randomized trials are currently not available. This route of administration can be a way to optimize the treatment, reducing the risk of colonization, especially in the first year, with a limited risk of adverse reactions [29].

Even if not confirmed by multivariate analyses, our data revealed a lower 30 days colonization and infection rates (overall and by MDR bacteria) in patients receiving standard systemic antibiotic prophylaxis (extended spectrum cephalosporin plus a glycopeptide) in comparison with atypical regimens, endorsing the clinical relevance to follow guidelines indications [38], in absence of alternative clinical needs.

We also found a significant link between the occurrence of pneumonia in the early post-transplant period and body weight of the recipients. A higher baseline BMI was associated to a reduced risk of developing pneumonia. Our finding could be explained by the fact that a major reservoir of lean and fat mass could permit to bear major stresses, such as the surgery, shortening the ICU stay, and supporting recovery period. In addition, underweight, as well as obesity, can lead to recovery problems during post-surgery period and can be linked to a partially harmed immunity function. Our findings are in line with previous reports [30]: a major incidence of post-LT infections was observed in underweighted patients, together with a shorter survival. Optimal baseline BMI seems to be associated with lower mortality rates, especially at 90 days and 1 year [31]. Underweighted and obese patients also seem to be at higher risk of poor outcome in the first year [32]. Obesity of grades II and III, on the other hand, is an absolute contraindication to transplant, while a less strict approach is adopted with mild obesity (grade I) in which transplant can be considered as an option [33]. An ideal baseline body weight is an important factor to estimate infectious risk and to assess the predicted outcome of lung recipients [30].

We found an association between baseline IPF and the development of pneumonia by MDR bacteria. Previous studies reported high rates of pulmonary and bloodstream infection in person affected by pulmonary fibrosis [34, 35]. In our cohort, patients with baseline IPF were more prone to develop respiratory infections than patients with COPD. This could be related to a higher pre-LT immunosuppression: before 2014 steroidal and immunosuppressive drugs (e.g., azathioprine) were the only therapy for IPF. Therefore, a greater cumulative exposure to these drugs could have been linked to the high incidence of comorbidities and infections. This observation could also partly explain the higher post-transplant mortality in IPF patients, which some authors highlighted [4].

A higher incidence of infections by bacteria harboring MDR is known after solid organ transplant [13, 36]. An active surveillance program during the pre- and post-transplant period including surface swabs and respiratory secretions culture is fundamental to steadily detect colonizing MDR bacteria, thus optimizing post-transplant antibiotic strategies when needed, particularly in patients with PGD. The cause of PGD is likely multifactorial with brain death–related donor lung injury, ischemia-reperfusion injury, infection, and cardiopulmonary bypass representing some of the possible etiologies. Moreover, infections can represent factors that may confound and/or amplify a diagnosis of primary graft dysfunction [15]. In our study, we observed a significantly higher prevalence of PGD (all grades) in MDR infected patients (94.7% versus 63.4% in non-MDR infected patients, *p* = 0.01).

Our analysis of independent risk predictors identified also a role of pre-transplant respiratory colonization and infection in predicting 30 days pneumonia (respectively AOR 5.43, 95% CI 1.03–28.54, *p* = 0.04, AOR 22.42, 95% CI 2.22–269.20, *p* = 0.01), highlighting the role of an accurate pre-transplant microbiological monitoring strategy. Either in pre- and post-transplant period an appropriate use of antibiotics (on the basis of antimicrobial susceptibility testing) is crucial, in order to both prevent possible adverse effects and avoid selective pressure on resistant bacteria [36, 37].

In conclusion, it is well known that anamnestic, clinical, and microbiological pre-transplant features are key factors for planning the post-transplant strategies. Recent guidelines [38] have focused on surgical site infection, but in LT an increasing infectious risk is obviously present for respiratory tract infections. Identification of high-risk patients before the LT could guide to personalize prophylaxis and therapy, when needed, and to lead to shorter and tailored antibiotic treatment, reducing adverse effects and selection of MDR strains. On the basis of our results, the risk of post-transplant lung infectious complications could be stratified by considering several factors such as the indication for LT, the type of LT, the presence of certain comorbidities, and the microbiologic assessment before LT. Importantly, our results suggest that a wider use of early nebulized therapies could be useful to prevent potentially harmful colonization from MDR strains, thus potentially lowering infectious risk.

## Supplementary Information

ESM 1 (PDF 68 kb)

## Data Availability

The datasets used and/or analyzed during the current study are available from the corresponding author on reasonable request.
