# Supplementary material for: Incidence and risk factors for respiratory tract bacterial colonization and infection in lung transplant recipients
Source: Eur J Clin Microbiol Infect Dis. 2021 Jan 21;40(6):1271–82. doi: 10.1007/s10096-021-04153-1 (PMC8139905; doi:10.1007/s10096-021-04153-1)
Supplement: Supplementary file 1 — (PDF 68 kb) [file 10096_2021_4153_MOESM1_ESM.pdf]

Supplementary Table 1. Risk factors for bacterial respiratory colonization 30-days after LT. Comparison between Not colonized, colonized by susceptible strains (SS) or by MDR bacteria.

Risk factors	No 30 days colonization N=63 (%)	30 days SS colonization N=21 (%)	30 days MDR colonization N=10 (%)	*p-value*
**Males**	38 (60.3)	14 (66.7)	7 (70)	0.770
**Median age** (IQR)	58 (52-62)	53 (48-56.5)	59 (54.8-64.3)	**0.031**
**Pre-LT BMI**	24 (20-28)	24 (20-29.1)	25.4 (20-30.5)	0.680
**Indication for transplant:**				
- **IPF**	26 (41.3)	7 (33.3)	8 (80)	0.156
- **COPD**	16 (25.4)	7 (33.3)	1 (10)	
- **Other**	21 (33.3)	7 (33.3)	1 (10)	
**Type of transplant:**				
- **Single**	40 (63.5)	5 (23.8)	7 (70)	0.004
- **Double**	23 (36.5)	16 (76.2)	3 (30)	
**Comorbidities** :	52 (82.5)	18 (85.7)	8 (80)	0.913
- **Osteoporosis/osteopenia**	30 (47.6)	13 (61.9)	5 (50)	0.524
- **Peptic disease**	5 (7.9)	2 (9.5)	4 (40)	**0.013**
- **Chronic coronary heart disease**	3 (4.8)	4 (19)	2 (20)	0.077
- **Diabetes**	16 (25.4)	4 (19)	3 (30)	0.768
- **Obesity**	12 (19)	4 (19)	4 (40)	0.310
- **Nephropathy**	2 (3.2)	1 (4.8)	0 (0)	0.780
**Hospitalization 90 days before LT**	14/59 (23.7)	4 (19)	2/10 (20)	0.683
**ICU admission 90 days before LT**	3/57 (5.3)	1/20 (5)	0/9 (0)	0.913
**Antibiotic treatment 90 days before LT**	14/56 (25)	2/20 (10)	1 (10)	0.319
**Baseline respiratory bacterial colonization (recipient):**				
- **Overall**	9 (14.3)	3 (14.3)	1 (10)	0.933
- **MDR bacteria**	2/61 (3.3)	0/20 (0)	0/9 (0)	0.742
**Baseline respiratory bacterial infection (recipient):**				
- **Overall**	4/59 (6.7)	1 (4.8)	1 (10)	0.668
- **MDR bacteria**	1/59 (1.7)	0 (0)	0/9 (0)	0.687
**Baseline respiratory bacterial colonization (donor):**				
- **Overall**	12 (19)	8 (38.1)	3/8 (37.5)	0.418
- **MDR bacteria**	1/50 (1.9)	1/18 (5.5)	0/8 (0)	0.876
**Baseline respiratory bacterial infection (donor):**				
- **Overall**	6/54 (11.1)	0/19 (0)	0/8 (0)	0.198
- **MDR bacteria**	0/50 (0)	0 (0)	0/8 (0)	0.512
**Post LT systemic antibiotic prophylaxis**	55 (87.3)	16 (76.2)	7 (70)	0.258
- **Standard**[a]	8 (12.7)	5 (23.8)	3 (30)	
- **Atypical**				
**Basiliximab induction**	20 (31.7)	11 (52.4)	3 (30)	0.21
**Immunosuppressive regimen:**				
**-Tacrolimus**	18/55 (32.7)	8/19 (42.1)	3/9 (33.3)	0.83
**-Cyclosporine**	35/55 (63.6)	11/19 (57.9)	6/9 (66.7)	
**-Other**	2/55 (3.6)	0/19 (0)	0/9 (0)	
**Aerosol prophylaxis,**	55 (87.3)	17 (81)	10 (100)	0.332
**length of days median (IQR)**	29 (21-44)	29 (22-40)	17 (6.5-31.5)	0.071

**Rejection**	17 (27)	6 (28.6)	3 (30)	0.975
**Days from LT, median (IQR)**	22 (17-37)	24 (12-31)	43 (20-50)	0.493
**PGD**	43 (69.4)	14 (66.7)	7 (70)	0.970
**Lenght of stay, days median (IQR)**	37 (29-66)	34 (28-61)	36.5 (28-61.3)	0.666

BMI body mass index; IPF Idiopathic pulmonary fibrosis; COPD chronic obstructive pulmonary disease; LT lung transplant; MDR multi-drug resistant; PGD Primary Graft Disfunction; [a] Comprehending one of each of the following classes: anti-pseudomonal cephalosporin (namely ceftazidime), anti Gram positive drug (namely vancomycin), antifungal prophylaxis (voriconazole) and trimethoprim-sulfamethoxazole
